# Fatuity of Old Age

**Published:** 1854-04

**Authors:** 


### FATUITY OF OLD AGE.

It is scarcely possible to conceive of a more terrible calamity than to be old and have no mind, not even the intelligence to know your own child! Mumblings and mutterings, ceaseless and incoherent. To have no understanding beyond that of the animal,—to eat, and drink, and sleep,—dead to yourself, and more than dead to all your kindred. It may come by degrees, it may come like the lightning's flash. It was told me once of one of the greatest clerical minds in America, that in the midst of a discourse, in which the whole congregation was wrapt with an intensity of attention, scarcely to be paralleled, for his was a giant mind among giants, he suddenly placed his hand upon his forehead, and bowing it forward, exclaimed, "God, as with a sponge, has blotted out my mind."

This fatuity is called in medical language, *mollities cerebri*,—softening of the brain. It is hopelessly incurable. M'Duffie and "Tom Moore" thus perished. It may be instructive to the general reader to know who are most liable to this terrible calamity. The great general cause of fatuity is a want of proper proportion of physical and mental exercise. The class which furnishes the largest number of such unfortunates, is that which thinks much and works but little, such as Clergymen, lawyers, poets, married people who have no children, married people who do not keep house but board out and have nothing to do, but live on their income. One of the greatest curses that can fall upon a man in this life is to be old and to be able to live without doing any thing, and thus living. If any woman reader of mine, over fifty, wishes to avoid fatuity, let her be careful not to place herself in a situation which will release her from the cares and duties of housekeeping. No man can say that he will not die fatuitous, despite of his iron constitution, who studies a great deal and devotes but little time to daily exercise; the only safeguard any student after fifty has against it is, that from youth to the hour of his death he shall spend several hours every day in active bodily employment out of doors. Remember the only certain, the only infallible preventive of fatuity is daily physical exercise from early life.

A recent letter writer says, "Glorious old Christopher North lies there in Scotland with the hand of death on him, and the cold, solemn shadow of the grave stealing over him. What merry bouts of wit we have had with him, and what ambrosial nights we have spent together! But the song is ended, the laughter is hushed, and the lord of the feast takes his departure. He has run his career, and that magnificent presence of his is now a wreck. The mountain that we used to look up to with such expectancy, is cold and dark, and will emit no more flashes of wit, or passion, or pathos. Wilson is one of those spirits who start in the race with the loftiest aspirations and boldest prognostications, armed at every point, and mighty to overcome. They loom upon us with such large proportions, and such a flush of glory clings about them, they seem like the early gods, looming upon us through the dawnlight of time. But somehow, they do not reach the goal of our prophecy. The flower of their promise dies out, but the fruit does not follow. The wondrous impression of their powers which they produce upon their immediate circle of friends and admirers does not get stamped upon the world, and the public reputation of Christopher North bears no comparison to that estimate of his genius entertained by his lovers and worshipers. They are apt to speak of what he might have done, we have to judge of what he has actually accomplished. They stand on the summit of their admiration, and speak of a land of promise we cannot see, and their report to us seems exaggerated. John Wilson has lived his life, rather than written it. Richly endowed, he has lavished his precious gifts in so many ways, rather than concentrate them in one, and no man can be the perfect master in all.

